# Dystonia Management: What to Expect From the Future? The Perspectives of Patients and Clinicians Within DystoniaNet Europe

**DOI:** 10.3389/fneur.2021.646841

**Published:** 2021-06-03

**Authors:** Marenka Smit, Alberto Albanese, Monika Benson, Mark J. Edwards, Holm Graessner, Michael Hutchinson, Robert Jech, Joachim K. Krauss, Francesca Morgante, Belen Pérez Dueñas, Richard B. Reilly, Michele Tinazzi, Maria Fiorella Contarino, Marina A. J. Tijssen, The Collaborative Working Group

**Affiliations:** ^1^Expertise Centre Movement Disorders Groningen, Department of Neurology, University Medical Centre Groningen, Groningen, Netherlands; ^2^Department of Neurology, Istituto di Ricovero e Cura a Carattere Scientifico Humanitas Research Hospital, Milan, Italy; ^3^Dystonia Europe, Brussels, Belgium; ^4^Neuroscience Research Centre, Institute of Molecular and Clinical Sciences, St George's University of London, London, United Kingdom; ^5^Institute of Medical Genetics and Applied Genomics and Centre for Rare Diseases, University of Tübingen, Tübingen, Germany; ^6^Department of Neurology, St. Vincent's University Hospital, Dublin, Ireland; ^7^Department of Neurology and Centre of Clinical Neuroscience, First Faculty of Medicine, Charles University, Prague, Czechia; ^8^Department of Neurosurgery, Medizinische Hochschule Hannover, Hanover, Germany; ^9^Department of Clinical and Experimental Medicine, University of Messina, Messina, Italy; ^10^Pediatric Neurology Research Group, Hospital Vall d'Hebron–Institut de Recerca (VHIR), Barcelona, Spain; ^11^School of Medicine, Trinity College, The University of Dublin, Dublin, Ireland; ^12^Department of Neuroscience, Biomedicine and Movement Science, University of Verona, Verona, Italy; ^13^Department of Neurology, Leiden University Medical Centre, Leiden, Netherlands; ^14^Department of Neurology, Haga Teaching Hospital, The Hague, Netherlands

**Keywords:** dystonia, DystoniaNet, collaboration, unmet needs, European network

## Abstract

Improved care for people with dystonia presents a number of challenges. Major gaps in knowledge exist with regard to how to optimize the diagnostic process, how to leverage discoveries in pathophysiology into biomarkers, and how to develop an evidence base for current and novel treatments. These challenges are made greater by the realization of the wide spectrum of symptoms and difficulties faced by people with dystonia, which go well-beyond motor symptoms. A network of clinicians, scientists, and patients could provide resources to facilitate information exchange at different levels, share mutual experiences, and support each other's innovative projects. In the past, collaborative initiatives have been launched, including the *American Dystonia Coalition*, the *European Cooperation in Science and Technology (COST—*which however only existed for a limited time), and the Dutch *DystonieNet* project. The European Reference Network on Rare Neurological Diseases includes dystonia among other rare conditions affecting the central nervous system in a dedicated stream. Currently, we aim to broaden the scope of these initiatives to a comprehensive European level by further expanding the DystoniaNet network, in close collaboration with the ERN-RND. In line with the ERN-RND, the mission of DystoniaNet Europe is to improve care and quality of life for people with dystonia by, among other endeavors, facilitating access to specialized care, overcoming the disparity in education of medical professionals, and serving as a solid platform to foster international clinical and research collaborations. In this review, both professionals within the dystonia field and patients and caregivers representing Dystonia Europe highlight important unsolved issues and promising new strategies and the role that a European network can play in activating them.

## Introduction

Dystonia is a movement disorder characterized by sustained or intermittent muscle contractions causing abnormal, often repetitive, movements, postures, or both (1). Besides motor symptoms, dystonia syndromes also include several non-motor symptoms, with an independent significant impact on health-related quality of life (2–8).

Dystonia has remained a rather enigmatic disorder despite it being the third most common movement disorder after parkinsonism and tremor and despite its major impact on health. One key problem, which remains today, is the multiplicity of causes of dystonia. This has meant that the traditional approach of looking for a unified pathophysiological model for a disorder, from which one can develop diagnostic biomarkers and treatments, has been difficult to apply. The possibility that pathophysiological and treatment efficacy studies are contaminated by inclusion of people with dystonia of differing etiologies is a real one and is perhaps an important reason why progress in treatment development has been slow.

Over the past 1–2 decades, gradual progress has been made in understanding the genetic underpinnings of some forms of dystonia, allowing the prospect of studying genetically defined cohorts of patients. In addition, pathophysiological studies have become more sensitive to the possibility of combining multiple etiologies of dystonia, alongside advances in identifying important subgroups of people with dystonia with specific etiologies, for example, functional dystonia. The wide spectrum of symptoms in dystonia has also been recognized, including its impact on mental health and cognitive and sensory processing. Treatment advances have been made, particularly in the successful use of deep brain stimulation (DBS) for certain types of dystonia, with intracranial recordings performed during these surgeries providing a novel source of pathophysiological data.

However, many gaps in knowledge remain, and efforts have been made in recent years to build networks that foster international research collaboration. One recent EU-focused infrastructural initiative is the European Reference Network (ERN) for Rare Neurological Diseases (RND), born with the aim to improve quality of life for RND patients and to facilitate the exchange of knowledge between healthcare professionals across borders (9). A patient can be virtually presented to a specialist in another country, with the aim to provide the best medical care without the need to travel. Moreover, it facilitates the collection of patient data, which is important for research purposes. The ERN-RND will interconnect tightly with DystoniaNet Europe as regards goals, PIs, and activities.

Specifically for dystonia, a research network was formed in 2011 by the European Dystonia Cooperation in Science and Technology (COST) Action. This action was aimed at promoting genetic studies, stimulating the development of experimental animal models, standardizing and harmonizing patient care, strengthening the scientific or medical expertise of young researchers and doctors through international exchanges between European research laboratories and expert centers, and educating the public and professionals about the disorder. The original workgroup included applicants from 18 European countries but later increased to encompass 24 European countries. There was also collaboration with the American Dystonia Coalition. Moreover, an important partner was Dystonia Europe, which is an umbrella organization for 22 national dystonia patient associations in 18 European countries, aiming at improving quality of life for people living with dystonia by focusing on the following: raise awareness, spread information, promote education and research, support lobbying and advocacy, and add value to the work of member associations.

Another collaborative multidisciplinary network was initiated in The Netherlands. In 2010, the Movement Disorders workgroup of the Dutch Neurological Society brought together several movement disorders specialists and physiotherapists to initiate *DystonieNet*. The main goal of this national network was to optimize cervical dystonia (CD) treatment by facilitating collaboration between experts, educating more healthcare providers, and promoting research. Another aim was to facilitate the patients' access to dystonia experts to quicken and improve diagnosis and treatment. In this context, a Dutch website (dystonia.net) was developed that serves as a platform for healthcare professionals. This website gives information on regional, national, and international meetings, focused skills workshops, and ongoing research studies on dystonia. A special feature on the website is the “Care Searcher” tool that lists all botulinum toxin units, movement disorder neurologists, or physical therapists specialized in dystonia, who can then be located by patients and clinicians by typing their zip code area. In addition, a newsletter is issued several times a year to spread the most recent news in the field of dystonia, and a special mobile phone application has been developed that provides information about the national guidelines for botulinum toxin treatment, as a handy tool in the outpatient clinic.

From the experience of the COST Network and the Dutch *DystonieNet*, the network was expanded to become *DystoniaNet Europe*. The aim of this European project is to expand the scope of the Dutch *DystonieNet* project to a European level. In the initial phase, Ireland and Slovakia joined the website project, and currently, more countries are in the process of joining. All the authors of this paper (see Authors/collaborative working group) represent countries, invited to be part of *DystoniaNet Europe*. Authors were selected based on their previous participation in European initiatives about Dystonia, but the participation in the Network is surely not exclusive. The topics of the paper were assigned to different members of the writing committee. A draft of the paper was shared with all the authors in the Authors/collaborative working group, who contributed important intellectual content.

The above-mentioned projects are major steps to provide answers to important knowledge gaps. In this paper, we will identify some of the unmet needs, not only both from the perspective of health professionals and researchers but also of the patients and caregivers across Europe. Patients' quotes, collected by DystoniaEurope, highlight the unmet needs shared by professionals and patients. We will also try to define the role that a European network such as *DystoniaNet* can have in facilitating the solution to these problems, as summarized in Table 1.

**Table 1:** Goals DystoniaNet Europe.

**Priorities of action of DystoniaNet Europe**	**Highlights**
**Diagnostic process**	
Improving dystonia awareness among patients and clinicians	•Organization of trainings for GPs, neurologists, physical therapists, with focus on diagnosis and treatment (for example botulinum toxin treatment).
Algorithm for diagnosis of dystonia	•Development of a diagnostic model for adult onset dystonia
Genetics	•Sharing of technical knowledge across gene banks •Pooling of data across existing genetic databases •Development of a European biobank
Neurophysiological diagnostic biomarkers dystonia	•Development of neurophysiological markers to discriminate dystonia from other movement disorders and to differentiate idiopathic, acquired, and functional dystonia •Gain knowledge of dysfunctional brain networks •Development of biomarkers for the effectiveness of DBS
Imaging biomarkers	•Gain knowledge about pathophysiological mechanisms •Development of automatic algorithms of image analysis
Endophenotypes	•Organization of large prospective studies of patients and relatives to prospectively study endophenotypes
**Treatment**	
Physical and occupation therapy	•Collection of evidence on different training programs and on cost effectiveness •Development of dystonia specific training programs, with a personalized approach •Collection of evidence about the effect of physical therapy and occupational therapy on pathophysiological mechanisms
Botulinum toxin treatment	•Collection of more evidence about some treatment aspects (e.g., use of polymyography and ultrasound in guiding injections) •Clinical studies aimed at improving the benefit/side effect ratio and reducing the number of non-responsive patients •Development of uniform European BoNT treatment guidelines •Improvement of access to treatment
Deep brain stimulation	•Development of an expert network across European countries for clinical consultation, sharing experiences, and outcomes •Development of European Dystonia DBS registry/biobank aimed to collect information about DBS outcomes in rare forms of dystonia and in children, rare side effects, and unexpected responses •Collection of evidence about biomarkers predictors of outcome
Non-motor symptoms	•Development of a non-motor symptom questionnaire for different dystonia subtypes •Improvement of treatment of non-motor symptoms •Development of a subjective goal outcome scale •Development of a multidisciplinary approach

## Diagnostic Process

### Improving Dystonia Awareness Among Patients and Clinicians

Currently, the diagnosis of dystonia is merely based on physical examination and recognition of patterns by an experienced clinician. This can be a diagnostic challenge due to a wide range of dystonia phenotypes and etiologies as well as dystonia mimics (such as functional dystonia). Dystonia can have different characteristics and can be the only symptom but also present as part of a mixed neurological and even systemic disorder (10). The etiological and clinical classification can be particularly difficult in childhood onset dystonias (11).

Due to the rarity of the disorder and the complexity of the presentation, there is often a significant delay in diagnosis. In CD, which is the most prevalent form of dystonia with well-defined clinical symptoms, the mean time from symptom onset to diagnosis varied in several studies from 3.7 (12) to 6.8 years (range, 0–53 years) (13) (for a patient's experience, see Box 1). Similar diagnostic delays were observed for other adult-onset focal and segmental dystonias, where in almost half of the cases, it took more than a year to reach the diagnosis (14).

Box 1 **Patient's experience.**

Dystonia patient: “Today I was diagnosed with dystonia after 18 years of having these awful shakes where everyone thinks I am saying no. I now have a name for it. It is not essential tremor, it is not in my mind, and it is a very real condition.”

Improvement of dystonia awareness and knowledge can be expected to enable patients to be referred more quickly to an experienced clinician and to obtain a correct diagnosis and addressing their symptoms more rapidly (for a patient's experience, see Box 2). The introduction of a nationwide Care Searcher tool could then help gain access to a more advanced diagnosis by a movement disorder specialist (including syndrome characterization and genetic profile) and to adequate treatment, including identifying patients who are candidates for more advanced treatments such as surgical therapies, including DBS. Besides that, in some countries (e.g., France), government actions have labeled Centers of Excellence for Rare Diseases, whose mission is to identify places of diagnosis and therapeutic expertise, as close as possible to patients and to ensure regional and national networks.

Box 2 **Patient's experience.**

Dystonia patient: “Much more needs to be done to raise awareness of this disease. General practitioners and nurses need to be educated on it. We get fed up hearing: “oh it is like Parkinson's then”.”

Education of medical professionals is one of the most important steps to achieve these goals. Here, a European network can play a role by the organization of training for general practitioners, general neurologists, and physical therapists. There is a need for a more structural practical education around dystonia diagnosis and treatment (for example, botulinum toxin workshops): these could be implemented also in the context of movement disorders curricula or fellowship programs for residents and young fellows, which are however lacking in some countries (15). In the COST initiative, three “dystonia schools” (Bol, Croatia; Groningen, the Netherlands; London, UK) were organized for young neurologists and scientists. In addition, awareness in the general population may be increased by means of media campaigns at a European level, involving national patient associations through Dystonia Europe. DystoniaNet, in close collaboration with the ERN-RND, can play a significant role in this.

### An Algorithm for the Diagnosis of Dystonia

Dystonia can be classified based on clinical characteristics (axis I, including age at onset, body distribution, temporal pattern, and associated features) and etiology (axis II) (1). Clinical characteristics form the basis for the etiological clinical suspicion and thereby give an indication of which supplementary laboratory tests or imaging should be performed.

The major developments in genetic testing, allowing us to analyze many genes in a relatively short time, make the case for a renewed diagnostic strategy: recently, a diagnostic algorithm has been proposed for dystonia occurring in children and adolescents (16). Similarly, we could envisage a diagnostic model for adults (see below). This should consider the availability of the diagnostic modalities in different countries and be open to continuous updates as knowledge increases and techniques become readily available and affordable. When a specific investigation (like genetic testing) is not available in one country, international collaboration may provide the solution to complete the diagnostic process. When all patients have undergone the same diagnostic process, larger groups of well-characterized rare dystonia subtypes can be collected, which would increase the statistical power of research at a pathophysiological and treatment level.

### Genetics

In 1997, the TorsinA (*TOR1A*) gene was the first to be identified as the major cause for young-onset (primary) generalized dystonia (17). However, monogenic causes of dystonia can only be found in 1–2% of the patients in an average dystonia clinic (18, 19). The other subgroup of dystonia with a genetic background includes the hereditary disorders in which dystonia is part of the symptom spectrum. This list contains isolated, combined, and complex dystonias. Importantly, some of the hereditary forms include treatable (metabolic) diseases (16).

In clinical practice, the possibility of testing for a genetic background of dystonia has evolved rapidly. With the development of next generation sequencing (NGS), it has become possible to analyze thousands of genes simultaneously. One of the NGS techniques involves targeted gene panel analysis, in which a specific set of preselected genes is tested. Compared with other techniques like whole-genome and whole-exome sequencing, the costs of a gene panel analysis are lower, and there are fewer spurious findings (20). The panel approach results in a higher percentage of confirmed molecular diagnoses than a more classical clinical approach based on diagnostic hypotheses. In addition, the average costs and the time needed to reach an etiological diagnosis are lower when gene panel analysis is used compared to single gene analysis (20, 21).

Genetic testing should be considered mainly in young onset dystonia patients, patients with a positive family history, patients with paroxysmal dystonia, and patients with other (neurological) symptoms (18, 20, 21). The benefits of testing include diagnostic certainty for the individual patient, which then can avoid further unnecessary investigations, information about the risk of recurrence in the family (22), and prevention of transmitting the affected gene to the next generation. This of course comes together with ethical issues that need to be addressed. Although rare, a genetic diagnosis may, in some cases, also alter treatment.

Gene panel analysis is likely to play an important role in the future. However, at this time, it is not uniformly available, and there are differences in patient selection, counseling, and gene panel composition across different countries. In addition, the rapidly expanding genetic findings require a systematic update of the genes included in the gene panel. A uniform panel across Europe, with a centralized update system, could have major benefits. Knowledge could be exchanged across laboratories, and techniques for coverage and lab protocols can be shared and optimized. For research purposes, a uniform approach would make it possible to derive reliable epidemiological data across countries and to create large cohorts of patients and controls to test for variants of unknown significance. For this purpose, following the example of some national databases such as the German DysTract, bio banking at a European level could be pursued. Alternatively, a more open and easier sharing of regional or national gene banks would allow for a continuous actualization and implementation of clinical and genetic data. This would form the basis to identify new causative and disease-modifying genes or risk factors for dystonia and attribute clinical significance to variants of unknown pathogenicity for genes already identified. In the future, the discovery of more dystonia-related genes, and the unraveling of the highly complex network of cellular pathways, will eventually increase our understanding of the dystonia pathophysiology and hopefully create new treatment options (23). Moreover, a European network could facilitate the transfer of expertise and sharing of best practice can be implemented.

### Neurophysiological Diagnostic Biomarkers Dystonia

Electrophysiological and sensory perceptual studies have supported the view of dystonia as a disorder of network dysfunction involving the basal ganglia, thalamus, cerebellum, and sensorimotor cortices (24).

Despite major advances that have provided a better understanding of dystonia pathophysiology by means of different neurophysiological techniques (25–28), the diagnosis mainly relies only on clinical features. It would be a major advantage if (1) neurophysiological testing could reliably discriminate dystonia from other movement disorders and (2) could support the etiological diagnosis (idiopathic, genetic, or functional) (for a patient's experience, see Box 3). To date, neurophysiological studies have been inconsistent in differentiating idiopathic from functional (29, 30) or genetic dystonia (31). Only recently, a few studies found between-groups differences using different neurophysiological techniques in subjects with different etiologies of dystonia (32–35). It has been possible to distinguish between children with acquired isolated genetic or idiopathic dystonias using a corticomuscular, intermuscular, and sensory perturbation paradigm (32). In small sample size studies in adults, the blink reflex recovery cycle (33) and the paired associative stimulation protocol (used to test sensorimotor plasticity) (34, 35) have been effective in discriminating, respectively, cranial and limb functional dystonia from idiopathic dystonia. However, sensorimotor plasticity has been shown to be highly variable across subjects with different phenotypes of idiopathic dystonia and also within the same phenotype (36). In addition, although it has been hypothesized that different phenotypes of dystonia reflect altered processing at different levels of a dysfunctional brain network (37), there is only preliminary supporting evidence for CD (38).

Box 3 **Patient's experience.**

Dystonia patient: “A test to diagnose dystonia would help to reduce the time to a diagnosis and to increase its certainty.”

Confirmation by testing homogeneous cohorts of subjects is needed to define which neural networks may underlie different dystonic manifestations (tremor, tonic posturing, patterned movements), localization in different body parts, and associated non-motor symptoms, such as pain. Finally, as the discriminatory power at individual level for any of these neurophysiological paradigms have never been tested, it is crucial to design studies on large samples to test putative diagnostic biomarkers in idiopathic dystonia, which may aid the differential diagnosis primarily with functional dystonia, given the normality of structural neuroimaging in both conditions and the different therapeutic pathways.

From a methodological point of view, there are several technical challenges. One challenge is to investigate whether different components of the network are involved in generating the heterogeneous clinical picture of dystonia. Electrophysiological signals from the cerebellum have traditionally been viewed as inaccessible to magnetoencephalography (MEG) and electroencephalography (EEG). However, recent advances have allowed MEG and EEG to detect cerebellar activity using a high-resolution tessellation model of the cerebellar cortex constructed from repetitive high-field (9.4 T) structural MR imaging (39).

Transcranial magnetic stimulation (TMS) has been used extensively to study motor cortex physiology and plasticity in dystonia, as well as sensorimotor integration (25). However, reproducibility of results across studies has been difficult. Multimodal approaches integrating different neurophysiological techniques with neuroimaging are envisaged not only to support the diagnostic process (i.e., functional and genetic dystonias) but also to determine predictors of response to treatment, in particular DBS.

Another possible biomarker for the effectiveness of DBS in dystonia is intermuscular coherence analysis. In children, both idiopathic/genetic and acquired dystonia share an abnormal low-frequency intermuscular coherence, but their intermuscular coherence patterns respond differently to a sensory perturbation (32). In adult dystonia patients, low-frequency and beta band intermuscular coherence partly correlate with dystonia severity and improvement after DBS. This finding suggests that intermuscular coherence can function as a biomarker for DBS efficacy in dystonia, although confirmation in larger studies is needed.

Increased low-frequency activity (3–12 Hz) in the internal globus pallidus (GPi) of dystonia patients has also been reported as a potential biomarker (40) that is coherent with dystonic EMG discharges and correlates with symptom severity as assessed by dystonia rating scales in a large cohort of patients with CD (41). In patients that present predominantly with phasic components, DBS indeed decreases this pallidal low-frequency activity (42), and DBS contacts localized close to the highest low-frequency peak are clinically most effective (41), which could be useful for parameter selection for DBS or as a feedback signal for closed-loop stimulation in the future.

These specific neurophysiological tests require expertise and often need to be performed on expensive machines. This restricts research on neurophysiological markers to a few specialized centers. To this end, a European network such as DystoniaNet together with the ERN-RND could support the diffusion of a broader knowledge of specific techniques among neurophysiologists and the pooling of larger cohorts of patients for neurophysiological studies. Further developments in the neurophysiological field could aid the diagnostic process and form a powerful tool for guiding new treatment approaches with less side effects.

### Imaging Biomarkers

Morphological and functional imaging currently offers no reliable markers that can be used in the differential diagnosis of dystonic syndromes. Some notable exceptions are dystonia syndromes caused by neurodegenerative disorders and disorders associated with focal lesions or with metal accumulation in the basal ganglia such as neurodegeneration with brain iron accumulation (NBIA) and Wilson's disease.

For the other forms of dystonia, limited local changes in gray matter volume or thickness, subtle changes in the organization of white matter, and aberrant functional connectivity affecting large-scale networks can be detected only on a large group basis. Knowledge in this field is still relatively scattered due to high phenotype variability. Most imaging studies concern focal dystonias, and relatively little is known about generalized dystonic syndromes.

Imaging findings have contributed to the understanding of dystonia as network disorders (nexopathies, circuitopathies) (43). The weakness of all imaging studies is the fact that they cannot distinguish between cause and effect (44). Usual findings include frequent structural changes and hyper-/hypoactive connections involved in somatosensory perception and its integration into motor circuits (45–47). These are mainly the cortico-striato-pallido-thalamo-cortical pathway and the cerebello-thalamo-cortical pathway, the dysfunction of which is manifested in both focal and generalized dystonias (48). The first one, which involve connections from the basal ganglia and thalamus to the primary sensorimotor cortex, is hyperactive and less responsive to regulatory feedback stimuli from the cortex and subthalamus (44) and thus probably associated with well-known hyperexcitability of the motor cortex (49). The latter causes insufficient inhibition of the motor cortex via hypofunctional connection projecting from the cerebellum through the thalamus (50). Interestingly, local changes in the SM cortex correspond to the cortical representation of body segments affected by task specific dystonias (51–53).

The variability of morphometric findings, functional activity, and connectivity of the motor network largely depends on the genotype of dystonia and, to some extent, on the genotype/phenotype interaction. Basal ganglia volume and activity differ not only among different mutations (DYT-TOR1A, DYT-THAP1) (54) but also between DYT-TOR1A patients and DYT-TOR1A asymptomatic carriers (55). However, there is no universal imaging picture on which a genetic mutation could be predicted.

Resting-state functional MRI (fMRI) (as opposed to a task-based fMRI), has the advantage of not being contaminated with the executive or sensory component of the voluntary movement and has shown that the dystonic motor network is abnormally connected even at rest. In task-specific dystonia, changes in basal ganglia, primary sensory cortex, and premotor and parietal cortices have been shown (56, 57). In CD patients, increased connectivity of the putamen and its connections with the cortex and other basal ganglia partially normalize after botulinum toxin injections (44). In addition, CD patients who can temporarily relieve dystonia using a sensory trick showed reduced resting connectivity of the SM network and increased cerebellar connectivity while imagining this trick (58). Thus, findings in focal dystonia are, to some extent, variable but limited to sensorimotor circuits, which has also been confirmed by multimodal studies (51, 59).

Imaging studies in dystonia have already had some practical consequences both in supporting the differential diagnosis of dystonia and in predicting DBS effect.

A meta-analysis of the anatomical position of the active contacts of implanted DBS leads allowed for the construction of a probabilistic map associated with the clinical benefit of pallidal DBS. The sweet spot was located at the ventrolateral margin of the GPi and sub-pallidal white matter (60). The volume of tissue activates also quantitatively affected the structural and functional connectivity of the premotor and motor cortices, thalamus, supplementary motor area (SMA), and cerebellum, proving the remote effects of pallidal DBS in dystonia patients (61). These results indicated that imaging could be used for optimal targeting and even to inform stimulation parameter choice.

Furthermore, great hopes are placed on automatic algorithms of image analysis based on neural networks and machine learning. The automatic classification of resting-state fMRI has correctly detected patients with spasmodic dysphonia (SD) (62), CD (63), or alien-hand dystonia in corticobasal syndrome (64) with sufficient sensitivity and specificity. This approach seems promising also in the search for potential biomarkers predicting future clinical effects of DBS. For example, classification using a support vector machine based on the distribution of cortical atrophy within the associative, SM, and visuomotor areas resulted in 88% accuracy in estimating the pallidal DBS outcome in patients with segmental and generalized dystonia (65). The future use of these methods therefore seems promising.

Combining different techniques together, such as supervised machine learning applied to standard diagnostic brain MRI together with measuring central motor conduction times (CMCT) with transcranial magnetic stimulation (TMS), or -evoked potentials (SEPs) together with dystonia severity scales, can help counsel patients and families of dystonic children regarding the likely benefit of DBS in acquired dystonias as well as provide personal predictive and decision-making data using receiver operating characteristic (ROC) curves (66). This process applied internationally could rapidly build gene-specific and acquired disease-specific decision-making tools.

### Endophenotypes

Temporal discrimination, the ability to determine two sequential stimuli as separate in time, is disturbed in a number of basal ganglia disorders. Abnormal temporal discrimination is not specific for idiopathic and genetic (67) dystonia but can also be found in functional dystonia, albeit being produced by a different mechanism (68). It is, however, a highly sensitive measure with 97% sensitivity in the most common form of adult onset focal dystonia: cervical dystonia. It shows age- and sex-related penetrance in unaffected first-degree relatives, being found in ~50% of female first-degree relatives after the age of 40 years, indicating full (100%) penetrance; in male relatives, its penetrance is ~40% (69).

Accumulating evidence over the last 15 years has indicated that abnormal temporal discrimination is a mediational endophenotype in adult-onset dystonia. The features of mediational endophenotypes are as follows: (a) they are an expression of a genetic mutation, necessarily present prior to disease onset; (b) they reflect disease susceptibility and are not altered by disease expression or severity; and (c) they are more penetrant than the phenotype (70). Mediational endophenotypes, found both in CD patients and, importantly, in their unaffected relatives, may illuminate pathogenetic mechanisms not obvious from the motor phenotype.

Further support of this endophenotype is that, in unaffected relatives with abnormal temporal discrimination (compared to relatives with normal temporal discrimination), it is associated with increased putaminal volume (71), reduced putaminal activity (72), and reduced activation in the superior colliculus in response to a looming stimulus (73).

It is proposed that abnormal temporal discrimination indicates a disturbance in the system involved in covert attentional orienting, involving processing of salient environmental sensory stimuli through the superior colliculus. The midbrain covert attentional network captures changes in the environment potentially important for survival, which requires inspection and action. It is likely that impaired inhibition, caused by defective GABAergic mechanisms at the level of the synapse, underlies both abnormal temporal discrimination and dystonia.

It is also likely that non-motor symptoms in dystonia, like mood disorders and abnormal social cognition, are also driven by disrupted subcortical mechanisms of covert attention. Salient environmental stimuli include emotional threats (visual or auditory) and require emotional threat detection by the medial amygdala. Social cognition (74) integrates cognitive processes, such as the ability to follow eye gaze, share attention, and recognize emotion, to distinguish between self and others' intentions. There are preliminary studies indicating disordered basic social cognition in patients with adult onset dystonia (75–77). It is suggested that abnormal basic social cognition (to emotional face and voice stimuli) in patients with CD reflects disrupted subcortical processing in the collicular–amygdala pathway for threat detection (basic social cognition). This may be linked to heightened levels of anxiety and depression.

The results from different studies on social cognition in focal dystonia have been often contradictory; a recent large study assessing all four major social cognition dimensions found that participants maintained generally intact social cognitive abilities (78). The authors did note reduced recognition of facial expressions of fear; some patients with CD showed defective empathy. In another study, higher anxiety and depression levels were associated with better performance on an Facial Affect Naming task, suggesting that patients with CD might overactivate perceptual processing of social stimuli to compensate for baseline increases in anxiety levels and lowered mood (79). Most of the, admittedly limited, research shows little evidence of deficits in complex social cognition in adult onset focal dystonia, but basic social cognition, including emotion recognition in facial expressions and prosody, may be impaired; this requires further investigation.

Given the high penetrance of abnormal temporal discrimination in unaffected female relatives, it may be worthwhile to examine the prevalence of mood disorder and impaired social cognition in this population (in comparison to female relatives with normal temporal discrimination) and to follow them up prospectively.

In addition, this purpose can only be achieved by means of collaborative studies collecting large populations of patients and their relatives.

## Treatment Challenges

### Motor Symptoms

Nowadays, the treatment of dystonia consists of several possible strategies, depending on the age of the patient, dystonia subtype, or other specific factors. The effect of treatment can be monitored by several motor scales, although they frequently fail to observe small effects. In general, oral medication such as anticholinergics, physical therapy, botulinum toxin (BoNT) injections, or surgical treatment including DBS or ablative procedures can be considered. Here, in cooperation with the ERN-RND, we will focus on the need for European guidelines for physiotherapy/occupational and BoNT therapy and the unmet needs for DBS.

#### Physical and Occupational Therapy

A specific physical therapy (PT) intervention for CD has been described by JP Bleton (80). It aims to strengthen the non-dystonic antagonist muscles and to learn or relearn motor skills. A recent single-blinded randomized controlled trial investigated the effectiveness of a specialized PT program on disability in CD, compared to a regular PT program (81). Both groups showed a significant improvement of motor symptoms after 12 months of treatment, but no difference between groups was found, as both programs were effective. However, the specialized therapy group showed significant improvement in general health perception and self-perceived improvement over the general therapy group (for a patient's experience, see Box 4). Importantly, total health-related costs were lower in favor of the specialized therapy group.

Box 4 **Patient's experience.**

Dystonia patient: “I have had cervical dystonia for at least 2 years and was treated with botulinum toxin with little effect. When I finally came to a physical therapist specialized in dystonia, it was the turning point. From her I got tools such as special exercises and advice on how to manage my dystonia. It was nice to start to feel some control again. My family has also witnessed how much happier I was after I started seeing the physical therapist.”

In the Netherlands, several physical therapists were trained for this study and continued treating patients with the specialized therapy after the successful results. Currently, it is crucial to widely spread the knowledge to physiotherapists across Europe and to provide them with adequate training. To this end, international training schools and active professional networks should be organized to promote exchange of experiences and the implementation of standardized physiotherapy programs across Europe.

This could result in an improvement of treatment with a reduction in motor symptoms and lower costs (for a patient's experience, see Box 5). Moreover, considering that many patients still consult a physical therapist first after the onset of dystonia symptoms, the delay in diagnosis could be improved.

Box 5 **Patient's experience.**

Dystonia patient: “I have been suffering from cervical dystonia for 14 years. I am receiving botulinum toxin treatment. A few years ago my neurologist referred me to physiotherapy at a local hospital in the town where I live. On the first appointment, it turned out that my physiotherapist had never heard of dystonia, but said she would try to help me. She was stretching my muscles for an hour. I did not want to risk worsening my condition and did not continue this therapy. As far as I know there is a lack of physiotherapists in my country who are familiar with dystonia and can help patients with this condition.”

Recently, a cognitive orientation for occupational therapy (COOP) approach has been successfully studied in children and adolescents with acquired and genetic dystonias, who, after DBS were not achieving their goals (riding a bicycle, applying mascara, catching and throwing balls, swimming, feeding, carrying, and pouring drinks) (82). COOP, previously used in stroke rehabilitation in adults with developmental coordination disorder, was shown effective in one study focused on three participant-selected goals. The trained COOP skills were transferable to two additional untreated goals, and the result was obtained over 10 1-h sessions compared to hundreds of hours of “conventional therapy practice” sessions in the past. Extension of this technique with multiple therapists has been studied, and the application of COOP to children and young people without DBS is now required.

The search for the most effective physical treatment program for dystonia patients is far from over. There is a need for different approaches for different kinds of dystonias, in both children and adults, taking into consideration the affected body region, the symptoms severity, the presence of comorbidities, and age, social life, and skills of the patients. Ideally, every patient should receive a personalized approach (such as COOP), based on the experience of the physiotherapist and the patient preferences.

To this end, new studies should be designed with sufficiently large populations, which would require multicenter efforts.

Future research should also focus on the effect of PT and OT on the pathophysiological mechanisms of dystonia and how this relates to the maladaptive neuroplastic changes (83). This would improve the understanding of pathophysiological mechanisms and possibly improve therapeutic strategies. Finally, a broad training program for physiotherapists should ideally rely not only on solid evidence of efficacy but also on data about feasibility and cost effectiveness: such studies are currently scarce.

#### Botulinum Toxin Injections

BoNT injections are the most important treatment choice for focal dystonias but can also be used in segmental or generalized dystonias to relieve symptoms. Extensive research resulted in class I evidence to support efficacy and safety of several BoNT formulations (83). Up to 70–85% of CD patients report a significant benefit on the motor symptoms but also on pain and quality of life (83).

However, there are still uncertainties, such as the optimal starting dose, the interval between injections, or the need for single or multipoint injections in dystonic muscles. Especially after long-time treatment, neutralizing antibodies can develop, with a negative effect on BoNT efficacy (84, 85). In addition, dystonia syndromes with tremor may require a different approach, which needs further investigation. The use of polymyography seems to be effective in guiding injections and improving patient satisfaction but needs confirmation in larger studies. In addition, the use of ultrasound to target muscles and reduce the episodes of dysphagia seems to be a promising option to improve botulinum toxin treatment (83, 86).

Uniform European BoNT treatment guidelines could improve treatment for patients and enable further research toward improving the benefit/side effect ratio of BoNT treatment and reducing the number of primary and secondary non-responsive patients. A standardized working definition of non-responsiveness should be developed, and dose finding and comparative studies across different BoNT toxins should be performed. In addition, the additional value of polymyography and ultrasound should be examined.

Another important need is to improve access to treatment uniformly. It is currently unclear how many patients who are candidates for treatment are not receiving it. Factors that explain under-referral should be investigated and addressed, including lack of knowledge among treating physicians, costs, and scarce availability of BoNT centers in some areas (15). The development of a multidisciplinary consultation were the patient visit the movement disorder specialist, directly followed by polymyography and treatment with BoNT, can also significantly improve the diagnostic and treatment process for the individual patient.

#### Deep Brain Stimulation

Dystonic symptoms can severely impair the patients quality of life, while the response of dystonia to oral medical treatment may be disappointing (87). DBS has been applied for different forms of dystonia since the late 1990s (88–90). Satisfactory results can be safely achieved in most patients—including very young children (91) who go through rigorous selection to identify and characterize specific types of dystonia known to benefit most from DBS (92–94) (for a patient's experience, see Box 6).

Box 6 **Patient's experience.**

Dystonia patient: “Recently I have had deep brain stimulation and right now I can look at you straight, so I feel amazing! My life … it is like being reborn … it is crazy … I can wake up, I can go to work, I can drive my car, I can do shopping, I can go around, I can go to a bar, I can talk to people. Last night I was in the bar here and I talked to everybody, whereas before that never happened. My confidence is back.”

DBS is a complex therapy that requires a team of highly specialized allied health professionals, neurologists and neurosurgeons, specific technical equipment, and expensive implantable materials. DBS management requires an intensive follow-up after surgery. This therapy may present with complications and rare and poorly understood side effects that need to be recognized and handled. In addition, the DBS field advances quickly, as new technological tools arrive on the market (95). The complexity of DBS treatment increases when different types of dystonia are concerned due to the availability of different targets (GPi, different thalamic targets, subthalamic nucleus), the wide range of possibilities with advanced stimulation options, and the variable response that can be observed. It is also worth mentioning that neurosurgical treatment of dystonia is not restricted to DBS alone but includes other options such as stereotactic lesioning, MRgFUS, or selective peripheral neurosurgery, which can be combined or may be proposed as an alternative or even as a rescue treatment in selected cases (96–100).

Unfortunately, not all patients experience the optimal benefit from DBS, and the response of different forms of dystonia, some of which are very rare, still needs to be adequately investigated. Finally, when severe forms of generalized dystonia needing surgical treatment concern children, the range of skills and expertise required becomes even wider (101, 102).

Regarding these considerations, it is evident that DBS for dystonia can only be offered in selected specialized centers. The geographical distribution of such centers is not uniform across Europe: some countries have no center at all (15), and some DBS centers do not treat dystonia patients or have a low volume of surgeries due to the lack of resources or qualified personnel. As a result, patients in some areas currently do not have access to this effective treatment (103) (for a patient's experience, see Box 7).

Box 7 **Patient's experience.**

Dystonia patient: “My doctor had even no idea that you can get deep brain stimulation for dystonia or whether that was a good option for me or not. Even when I asked to be referred he wouldn't know where to refer me to.”

Moreover, while DBS centers with significant experience in this field can encounter difficulties in patient selection and postoperative management, such problems apply even more so to centers with less experience or smaller annual volume.

An expert network across European countries, gathering regularly in (virtual) meetings, could provide the needed infrastructure for clinical consultation in relation to challenging cases, exchange of experiences with the prevention and management of complications, and sharing of outcomes for the rarest forms of dystonia undergoing surgery. The virtual consultation infrastructure of ERN-RND as well as bilateral agreements between centers of different countries within the network could facilitate referral of patients to centers with specific expertise for treatment.

Such an initiative could have an immediate impact on daily practice, but it could also form the basis for the institution of a European Dystonia DBS registry, as it has been implemented already in some countries at a national level for special forms of dystonia in children (104). The registry could serve to collect information about DBS outcomes in rare forms of dystonia and in children, rare side effects, and unexpected responses.

At a subsequent stage, it could be supplemented with infrastructures for biobanking. Indeed, although a growing amount of data suggest that some patient characteristics may inform patient selection for surgery (105–108), at the moment, there are only tentative clinical, neuroradiological, genetic, or neurophysiological elements that could predict individual surgery outcome [as described above (66)]. Such much-needed biomarkers need to be rolled out across a wider population and pooled diagnostic subgroups, and this requires a collective effort, where different centers would not only contribute clinical data but also share infrastructures and expertise in the different fields.

### Non-motor Symptoms

Recently, the importance of non-motor symptoms (NMS) associated to dystonia has been brought to light. The lifetime prevalence of psychiatric disorders can reach up to 91.4% in CD patients and mainly consists of depressive symptoms and anxiety disorders (3, 109). Besides psychiatric disturbances, other NMS such as fatigue, sleep disorders, and pain are also highly prevalent (4).

Recognition and correct evaluation of the NMS associated with dystonia is of paramount importance for the choice of treatment approaches that would also target this important aspect (for a patient's experience, see Box 8).

Box 8 **Patient's experience.**

Mother of 7-year-old girl with dystonia: “We were on a waiting list for a year and just recently we have started to go to regular psychologist meetings. I think it is so important for family, friends and professionals to be aware and to be educated that dystonia doesn't just affect people physically.”

#### Non-motor Symptom Questionnaire

The high prevalence of NMS and the impact on the patients' well-being demands a more structural screening toward NMS during the regular outpatient visits. For this purpose, a standardized, validated NMS questionnaire specific for dystonia patients is needed to identify the symptoms and to evaluate the effect of treatment.

Recently, a novel 14-item self-completed questionnaire has been introduced (110). This Dystonia Non-motor Symptoms Questionnaire (DNMSQuest) covers seven domains including sleep, autonomic functions, fatigue, emotional well-being, stigma, activities of daily living, and sensory symptoms, and was tested in craniocervical dystonia patients. It appeared robust and easy to apply in daily practice, with just 14 questions that could be answered in about 5 min with yes or no. A possible disadvantage is that it does not score the severity of symptoms, for which additional information is required from the patient. Furthermore, it has only been validated for CD, so further validation in other dystonia subtypes is required. A European network would facilitate larger studies to create a questionnaire also for NMS severity and to validate the NMS questionnaire in other dystonia subtypes ll.

#### Treatment of Non-motor Symptoms

Treatment of NMS is important not only to improve the significant impact that they have on the quality of life but also to investigate their effect on motor symptoms and pathophysiological networks.

Psychiatric symptoms like depression and anxiety disorders are associated with neurotransmitter disturbances and are treated with medications influencing neurotransmitter systems like serotonin, dopamine, and noradrenalin. Importantly, these systems are involved in dystonia as well (111, 112). Safety profiles of most medications are based on a healthy (or non-dystonia) population and need further investigation in dystonia patients. One study showed that prescribing selective serotonin reuptake inhibitors (SSRIs) to CD patients is safe, with no deterioration of motor symptoms, but the effect on non-motor symptoms needs to be examined in larger studies investigating higher dose and longer schedule (113).

Evidence regarding the best treatment for NMS such as fatigue, sleep disturbances, and cognitive problems are still at an even more rudimentary stage. Besides pharmacological interventions, an approach including PT, cognitive therapy, coping strategies, and caregiver support, possibly in the context of a multidisciplinary rehabilitation program, could possibly contribute to a better well-being and requires further investigation.

An integrated treatment approach of motor and non-motor symptoms aiming at improving quality of life requires further research, also considering that the several dystonia forms have their own pathopsychological mechanisms and related NMS spectrum. Especially for the rare dystonia subtypes, this cannot be realized without international collaboration.

#### Subjective Goals as Outcome Measures

In general, the goal of any treatment is to improve quality of life and patient's satisfaction. To date, the standard way of assessing the effect of dystonia treatments is to measure the reduction in motor symptoms and, more recently, non-motor symptoms, which can be objectively measured. However, current scales are often not fully capable of accurately reflecting changes that are relevant for the patients. Indeed, small changes in predefined scores can make a big difference in daily life in some cases, while, on the other hand, measurable improvements do not always translate in significant ameliorations in functioning or independence.

A different approach could be to aim directly for functional improvement in daily life, as defined by the patients themselves (114, 115). One previous study examined the effectiveness of GPi DBS in dystonia patients on preoperatively set of functional priorities in daily living (116), measured with the Canadian Occupational Performance Measure (COPM). Priorities varied between patients but showed a significant improvement in performance and satisfaction after DBS in all. Importantly, improvement was reported both by the motor responders and by several patients classified as non-responders based on the motor outcome. Such an approach focuses on important improvements for the individual patient that would not have been objectified with a general motor or non-motor symptom rating scale only and could be applied also to other treatment such as BoNT, psychotherapy or PT, or even to potential new treatments being investigated in clinical trials (116, 117). A similar effect was also shown in other studies, were patients' perceptions in changes in life after DBS were studied with thematic interviews, instead of motor rating scales (118, 119).

#### A Multidisciplinary Approach

The diagnosis of dystonia motor symptoms, the recognition of the non-motor spectrum, and the identification of syndromes are very challenging. In addition, other factors can complicate the diagnostic process, like an abnormal development in children and the wide range of possible etiologies (120).

In other movement disorders, such as Parkinson's disease, a multidisciplinary approach has increasingly been shown to be beneficial (121). Also in children with movement disorders like dystonia, a multidisciplinary approach has already shown significant improvement in phenotyping, a high diagnostic yield and minimal diagnostic delay (120). Future research should investigate the additional value of a multidisciplinary approach also in the adult dystonia population.

The composition of a multidisciplinary team will vary at different levels. All patients may benefit from experienced Allied Health Professionals for support and specialized interventions (102) and application of the principles of the International Classification of Function (ICF). Children might require teams with a pediatrician, geneticist, or a specialist in metabolic diseases, although differences per center and per country can be expected. Adults may benefit from a multidisciplinary approach covering also the broad range of non-motor symptoms. A European network could advise on the specialists that would preferably be involved in the multidisciplinary team. When the optimal composition is not possible in a single center, multicenter collaboration can offer additional expertise. When highly specialized professionals are needed, international collaboration could be envisaged. In this way, multicenter collaborations could help overcome the shortcomings of the single centers, possibly also by implementing teleconsultation with external specialists.

For all dystonia patients, a dedicated multidisciplinary team may shorten the diagnostic delay, improve the classification and diagnostic yield, and play an important role in a timely and optimal treatment. In addition, genetic counseling may decrease the uncertainty for patients and families, not only concerning the cause of their symptoms but also about the consequence for next generations. Probably, this approach will result in reduced costs by reducing unnecessary investigations and, with a timely correct treatment, promoting faster participation in society.

Multicenter (international) collaborations pose several challenges, starting from identifying the correct specialists, allocating time, and solving technological communication issues while preserving data safety. The reality of the different countries with cultural and social differences, along with the geographical and technical disparities, must also be taken into account.

## Patient and Caregiver Perspective

As mentioned above, the route to dystonia diagnosis can be very challenging, causing prolonged suffering for many dystonia patients.

The impact of dystonia motor and non-motor symptoms is also reflected in the patients' stories Dystonia Europe receives. The boxes within the text provide a short insight into patients' experiences.

Based on the evidence and reports collected, which reflect the patients' and caregivers perspective, there are some goals in the care of dystonia patients that should be prioritized.

These include the following:

- Improving education and training for (young) neurologists and for general practitioners to speed up diagnosis and initiation of treatment;
- Promoting specialized dystonia centers across Europe with expertise in diagnosing and treating patients with more severe forms of the disease;
- Building up multidisciplinary teams, including, among others, neurologists, physiotherapists, occupational therapists, and psychologists for the care of dystonia patients;
- Training of physiotherapists and occupational therapists specialized in the treatment of dystonia patients and facilitating access to physiotherapy across Europe;
- Increasing awareness by developing standardized dystonia information material translated to the different European languages.

There are still major gaps in public understanding of dystonia and the psychological and financial burden that it may bring, in medical knowledge, and in timing of diagnosis and access to treatment for dystonia patients across Europe. To close these gaps and uniformly improve care and quality of life for dystonia patients, we need to work together: the medical profession, researchers, policy makers, patients, and carers. Therefore, the establishment of a strong international European dystonia network is much advocated by the dystonia community. The opportunity to collaborate across borders on education (with specialized dystonia training schools), research projects, improvement of dystonia awareness, etc. is the base to achieve the best care and improve quality of life for dystonia patients throughout Europe.

## Conclusions

Although dystonia is the third most common movement disorder after tremor and Parkinson's disease, it is still relatively rare. The wide heterogeneity of dystonia presentations makes it difficult to collect large numbers of specific dystonia subtypes for research purposes. Previous studies tended to lump patients with different forms of dystonia together (whether these were about diagnosis, pathophysiology, or treatment), while there are multiple different pathophysiological processes leading to different dystonia phenotypes. This may have affected the results.

DystoniaNet Europe is born with the aim of connecting dystonia experts and patients all over Europe in a network that can form the basis for leveling care at an upper level and for supporting large multicenter research project to advance knowledge. Such a network could also lay the foundations for a European registry to support future dystonia studies by the collection of data from different countries.

Leveraging existing infrastructure, we will be collaborating and interconnecting with the ERN-RND; however, we will also reach out to other networks such as the American Dystonia Coalition, aiming at a continuous fruitful interchange, which can enrich both associations and further advance knowledge through collaboration.

With joined forces, dystonia research can reach an important next level to further improve dystonia care and treatment.

## Collaborative Working Group

D. Arkadir, T. Baumer, K. Bhatia, N. Diederich, Y.P. Christou, C. Falup-Pecurariu, D. Georgiev, V. Han, S. Hassin -Baer, C. Klein, J.H.T.M. Koelman, A. Kuhn, S. Lalli, J.P. Lin, A. Lokkegaard, M.H. Martikainen, M.J. Marti, I. Milanov, P. Mir, M. Moller, E. Moro, S. O'Riordan, C. Painous, Z. Pirtosek, A. Pisani, A. Puschmann, A. Quartarone, M. Relja, D. Ruge, I.M. Skogseid, M. Skorvanek, A. Socher, M. Stamelou, A. Stepens, P. Taba, G. Tamas, A. Taravari, K. Valtteri, A. Valadas, W. Vandenberghe, M. Vidailhet, T. Warner, C. Wang, and G. Zorzi.

## Author Contributions

MS: organization, design, and writing the manuscript. AA, ME, and HG: manuscript reviewing and critique. MB, MH, RJ, JK, FM, RR, and MT: manuscript writing and critique. MC: design and writing the manuscript and critique. MAJT: design and manuscript reviewing and critique. All authors in the Appendix reviewed the manuscript for important intellectual content.

## Conflict of Interest

FM received speaking honoraria from Abbvie, Medtronic, Zambon, Bial, Merz; travel grants from the International Parkinson's disease and Movement Disorder Society; advisory board fees from Merz; Consultancies fees from Boston Scientific, Merz and Bial; research support from Boston Scientific, Merz and Global Kynetic; royalties for the book “Disorders of Movement” from Springer; member of the editorial board of Movement Disorders, Movement Disorders Clinical Practice, European Journal of Neurology. RR received grants from Science Foundation Ireland, Health Research Board, Dystonia Medical Research Foundation, Enterprise Ireland Commercialisation Fund, Disruptive Technologies Innovation Fund, IBM Faculty Award, Cochlear Research and Development Ltd, Healt Research Board and Dystonia Ireland, Enterprise Ireland Innovation Partnership with Vitalograph. JK is a consultant of Medtronic and Boston Scientific. RJ received consulting fees from Cardion, NeuroPa centrum, and Desitin. The remaining authors declare that the research was conducted in the absence of any commercial or financial relationships that could be construed as a potential conflict of interest.

## References

[1] Albanese A Bhatia K Bressman SB Delong MR Fahn S Fung VS. Phenomenology and classification of dystonia: a consensus update. Mov Disord. (2013) 28:863–73. 10.1002/mds.25475 23649720 PMC3729880

[2] Smit M Bartels AL Kuiper A Kamphuis ASJ Han V Tijssen MAJ. The frequency and self-perceived impact on daily life of motor and non-motor symptoms in cervical dystonia. Mov Disord Clin Pract. (2017) 4:750–4. 10.1002/mdc3.12510 30363474 PMC6174508

[3] Smit M Kuiper A Han V Jiawan VC Douma G van Harten B. Psychiatric co-morbidity is highly prevalent in idiopathic cervical dystonia and significantly influences health-related quality of life: results of a controlled study. Parkinsonism Relat Disord. (2016) 30:7–12. 10.1016/j.parkreldis.2016.06.004 27321988

[4] Smit M Kamphuis ASJ Bartels AL Han V Stewart RE Zijdewind I. Fatigue, sleep disturbances, and their influence on quality of life in cervical dystonia patients. Mov Disord Clin Pract. (2016) 4:517–23. Available online at: http://doi.wiley.com/10.1002/mdc3.12459 (accessed December 11, 2016). 30363425 10.1002/mdc3.12459 PMC6174405

[5] Conte A Berardelli I Ferrazzano G Pasquini M Berardelli A Fabbrini G. Non-motor symptoms in patients with adult-onset focal dystonia: sensory and psychiatric disturbances. Parkinsonism Relat Disord. (2015) 22:S111–4. 10.1016/j.parkreldis.2015.09.001 26360238

[6] Zurowski M McDonald WM Fox S Marsh L. Psychiatric comorbidities in dystonia: emerging concepts. Mov Disord. (2013) 28:914–20. 10.1002/mds.25501 23893448 PMC3842100

[7] Ben-Shlomo Y Camfield L Warner T group E collaborative. What are the determinants of quality of life in people with cervical dystonia? J Neurol Neurosurg Psychiatry. (2002) 72:608–14. 10.1136/jnnp.72.5.608 11971047 PMC1737851

[8] Skogseid IM Malt UF Røislien J Kerty E. Determinants and status of quality of life after long-term botulinum toxin therapy for cervical dystonia. Eur J Neurol. (2007) 14:1129–37. 10.1111/j.1468-1331.2007.01922.x 17708754

[9] European Reference Network. Neurological Diseases (2020). Available online at: http:/www.ern-rnd.eu (accessed November 1, 2020).

[10] van Egmond ME Contarino MF Lugtenberg CHA Peall KJ Brouwer OF Fung VSC . Variable interpretation of the dystonia consensus classification items compromises its solidity. Mov Disord. (2019) 34:317–20. 10.1002/mds.27627 30726575

[11] Lumsden DE Gimeno H Lin J-P. Classification of dystonia in childhood. Parkinsonism Relat Disord. (2016) 33:138–41. 10.1016/j.parkreldis.2016.10.001 27727009

[12] Tiderington E Goodman EM Rosen AR Hapner ER Johns MM III Evatt ML. How long does it take to diagnose cervical dystonia? J Neurol Sci. (2013) 335:72–4. 10.1016/j.jns.2013.08.028 24034410 PMC3840082

[13] Bertram KL Williams DR. Delays to the diagnosis of cervical dystonia. J Clin Neurosci Off J Neurosurg Soc Australas. (2016) 25:62–4. 10.1016/j.jocn.2015.05.054 26601813

[14] Macerollo A Superbo M Gigante AF Livrea P Defazio G. Diagnostic delay in adult-onset dystonia: data from an Italian movement disorder center. J Clin Neurosci Off J Neurosurg Soc Australas. (2015) 22:608–10. 10.1016/j.jocn.2014.09.014 25577433

[15] Tamás G Fabbri M Falup-Pecurariu C Teodoro T Kurtis MM Aliyev R. Lack of accredited clinical training in movement disorders in Europe, Egypt, and Tunisia. J Parkinsons Dis. (2020) 10:1833–43. 10.3233/JPD-202000 32651331

[16] van Egmond ME Kuiper A Eggink H Sinke RJ Brouwer OF Verschuuren-Bemelmans CC . Dystonia in children and adolescents: a systematic review and a new diagnostic algorithm. J Neurol Neurosurg Psychiatry. (2015) 86:774–81. 10.1136/jnnp-2014-309106 25395479

[17] Ozelius LJ Hewett JW Page CE Bressman SB Kramer PL Shalish C. The early-onset torsion dystonia gene (DYT1) encodes an ATP-binding protein. Nat Genet. (1997) 17:40–8. 10.1038/ng0997-40 9288096

[18] Zech M Jech R Boesch S Škorvánek M Weber S Wagner M. Monogenic variants in dystonia: an exome-wide sequencing study. Lancet Neurol. (2020) 19:908–18. 10.1016/S1474-4422(20)30312-4 33098801 PMC8246240

[19] MDSGene(2020). Available online at: MDSGene.org (accessed November 1, 2020).

[20] van Egmond ME Lugtenberg CHA Brouwer OF Contarino MF Fung VSC Heiner-Fokkema MR . A post hoc study on gene panel analysis for the diagnosis of dystonia. Mov Disord. (2017) 32:569–75. 10.1002/mds.26937 28186668

[21] Gorcenco S Ilinca A Almasoudi W Kafantari E Lindgren AG Puschmann A. New generation genetic testing entering the clinic. Parkinsonism Relat Disord. (2020) 73:72–84. 10.1016/j.parkreldis.2020.02.015 32273229

[22] Charlesworth G Bhatia KP Wood NW. The genetics of dystonia: new twists in an old tale. Brain. (2013) 136:2017–37. 10.1093/brain/awt138 23775978 PMC3692036

[23] Verbeek DS Gasser T. Unmet needs in dystonia: genetics and molecular biology-how many dystonias? Front Neurol. (2016) 7:241. 10.3389/fneur.2016.00241 28138320 PMC5237827

[24] Jinnah HA Neychev V Hess EJ. The anatomical basis for dystonia: the motor network model. Tremor Other Hyperkinet Mov (N Y). (2017) 7:506. 10.7916/D8V69X3S 29123945 PMC5673689

[25] Quartarone A Rizzo V Morgante F. Clinical features of dystonia: a pathophysiological revisitation. Curr Opin Neurol. (2008) 21:484–90. 10.1097/WCO.0b013e328307bf07 18607211

[26] Tinazzi M Fiorio M Fiaschi A Rothwell JC Bhatia KP. Sensory functions in dystonia: insights from behavioral studies. Mov Disord. (2009) 24:1427–36. 10.1002/mds.22490 19306289

[27] Bologna M Berardelli A. Cerebellum: an explanation for dystonia? Cerebellum Ataxias. (2017) 4:6. 10.1186/s40673-017-0064-8 28515949 PMC5429509

[28] Conte A McGovern EM Narasimham S Beck R Killian O O'Riordan S. Temporal discrimination: mechanisms and relevance to adult-onset dystonia. Front Neurol. (2017) 8:625. 10.3389/fneur.2017.00625 29234300 PMC5712317

[29] Espay AJ Morgante F Purzner J Gunraj CA Lang AE Chen R. Cortical and spinal abnormalities in psychogenic dystonia. Ann Neurol. (2006) 59:825–34. 10.1002/ana.20837 16634038

[30] Morgante F Tinazzi M Squintani G Martino D Defazio G Romito L. Abnormal tactile temporal discrimination in psychogenic dystonia. Neurology. (2011) 77:1191–7. 10.1212/WNL.0b013e31822f0449 21900627

[31] Edwards MJ Huang Y-Z Wood NW Rothwell JC Bhatia KP. Different patterns of electrophysiological deficits in manifesting and non-manifesting carriers of the DYT1 gene mutation. Brain. (2003) 126:2074–80. 10.1093/brain/awg209 12821514

[32] McClelland VM Cvetkovic Z Lin J-P Mills KR Brown P. Abnormal patterns of corticomuscular and intermuscular coherence in childhood dystonia. Clin Neurophysiol Off J Int Fed Clin Neurophysiol. (2020) 131:967–77. 10.1016/j.clinph.2020.01.012 32067914 PMC7083222

[33] Schwingenschuh P Katschnig P Edwards MJ Teo JTH Korlipara LVP Rothwell JC. The blink reflex recovery cycle differs between essential and presumed psychogenic blepharospasm. Neurology. (2011) 76:610–4. 10.1212/WNL.0b013e31820c3074 21321334 PMC3053342

[34] Quartarone A Rizzo V Terranova C Morgante F Schneider S Ibrahim N. Abnormal sensorimotor plasticity in organic but not in psychogenic dystonia. Brain. (2009) 132:2871–7. 10.1093/brain/awp213 19690095 PMC2997979

[35] Kojovic M Pareés I Kassavetis P Palomar FJ Mir P Teo JT. Secondary and primary dystonia: pathophysiological differences. Brain. (2013) 136:2038–49. 10.1093/brain/awt150 23771342

[36] Sadnicka A Hamada M. Plasticity and dystonia: a hypothesis shrouded in variability. Exp brain Res. (2020) 238:1611–7. 10.1007/s00221-020-05773-3 32206849 PMC7413892

[37] Latorre A Rocchi L Bhatia KP. Delineating the electrophysiological signature of dystonia. Exp Brain Res. (2020) 238:1685–92. 10.1007/s00221-020-05863-2 32712678

[38] Shaikh AG Zee DS Crawford JD Jinnah HA. Cervical dystonia: a neural integrator disorder. Brain. (2016) 139:2590–9. 10.1093/brain/aww141 27324878 PMC5840887

[39] Samuelsson JG Sundaram P Khan S Sereno MI Hämäläinen MS. Detectability of cerebellar activity with magnetoencephalography and electroencephalography. Hum Brain Mapp. (2020) 41:2357–72. 10.1002/hbm.24951 32115870 PMC7244390

[40] Silberstein P Kühn AA Kupsch A Trottenberg T Krauss JK Wöhrle JC. Patterning of globus pallidus local field potentials differs between Parkinson's disease and dystonia. Brain. (2003) 126:2597–608. 10.1093/brain/awg267 12937079

[41] Neumann W-J Horn A Ewert S Huebl J Brücke C Slentz C. A localized pallidal physiomarker in cervical dystonia. Ann Neurol. (2017) 82:912–24. 10.1002/ana.25095 29130551

[42] Barow E Neumann W-J Brücke C Huebl J Horn A Brown P. Deep brain stimulation suppresses pallidal low frequency activity in patients with phasic dystonic movements. Brain. (2014) 137:3012–24. 10.1093/brain/awu258 25212852 PMC4813762

[43] Warren JD Rohrer JD Hardy J. Disintegrating brain networks: from syndromes to molecular nexopathies. Neuron. (2012) 73:1060–2. 10.1016/j.neuron.2012.03.006 22445334 PMC3389343

[44] Brodoehl S Wagner F Prell T Klingner C Witte OW Günther A. Cause or effect: altered brain and network activity in cervical dystonia is partially normalized by botulinum toxin treatment. NeuroImage Clin. (2019) 22:101792. 10.1016/j.nicl.2019.101792 30928809 PMC6444302

[45] Obermann M Yaldizli O De Greiff A Lachenmayer ML Buhl AR Tumczak F. Morphometric changes of sensorimotor structures in focal dystonia. Mov Disord. (2007) 22:1117–23. 10.1002/mds.21495 17443700

[46] Draganski B Thun-Hohenstein C Bogdahn U Winkler J May A. Motor circuit” gray matter changes in idiopathic cervical dystonia. Neurology. (2003) 61:1228–31. 10.1212/01.WNL.0000094240.93745.83 14610125

[47] Etgen T Mühlau M Gaser C Sander D. Bilateral grey-matter increase in the putamen in primary blepharospasm. J Neurol Neurosurg Psychiatry. (2006) 77:1017–20. 10.1136/jnnp.2005.087148 16690695 PMC2077759

[48] Simonyan K. Neuroimaging applications in dystonia. Int Rev Neurobiol. (2018) 143:1–30. 10.1016/bs.irn.2018.09.007 30473192 PMC6331056

[49] Ridding MC Sheean G Rothwell JC Inzelberg R Kujirai T. Changes in the balance between motor cortical excitation and inhibition in focal, task specific dystonia. J Neurol Neurosurg Psychiatry. (1995) 59:493–8. 10.1136/jnnp.59.5.493 8530933 PMC1073711

[50] Argyelan M Carbon M Niethammer M Ulug AM Voss HU Bressman SB. Cerebellothalamocortical connectivity regulates penetrance in dystonia. J Neurosci. (2009) 29:9740–7. 10.1523/JNEUROSCI.2300-09.2009 19657027 PMC2745646

[51] Bianchi S Fuertinger S Huddleston H Frucht SJ Simonyan K. Functional and structural neural bases of task specificity in isolated focal dystonia. Mov Disord. (2019) 34:555–63. 10.1002/mds.27649 30840778 PMC6945119

[52] Egger K Mueller J Schocke M Brenneis C Rinnerthaler M Seppi K. Voxel based morphometry reveals specific gray matter changes in primary dystonia. Mov Disord. (2007) 22:1538–42. 10.1002/mds.21619 17588241

[53] Bianchi S Battistella G Huddleston H Scharf R Fleysher L Rumbach AF. Phenotype- and genotype-specific structural alterations in spasmodic dysphonia. Mov Disord. (2017) 32:560–8. 10.1002/mds.26920 28186656 PMC5578762

[54] Carbon M Eidelberg D. Abnormal structure-function relationships in hereditary dystonia. Neuroscience. (2009) 164:220–9. 10.1016/j.neuroscience.2008.12.041 19162138 PMC2760608

[55] Draganski B Schneider SA Fiorio M Kloppel S Gambarin M Tinazzi M. Genotype-phenotype interactions in primary dystonias revealed by differential changes in brain structure. Neuroimage. (2009) 47:1141–7. 10.1016/j.neuroimage.2009.03.057 19344776 PMC2741581

[56] Delnooz CCS Helmich RC Toni I van de Warrenburg BPC. Reduced parietal connectivity with a premotor writing area in writer's cramp. Mov Disord. (2012) 27:1425–31. 10.1002/mds.25029 22886735

[57] Battistella G Simonyan K. Top-down alteration of functional connectivity within the sensorimotor network in focal dystonia. Neurology. (2019) 92:e1843–51. 10.1212/WNL.0000000000007317 30918091 PMC6550502

[58] Sarasso E Agosta F Piramide N Bianchi F Butera C Gatti R. Sensory trick phenomenon in cervical dystonia: a functional MRI study. J Neurol. (2020) 267:1103–15. 10.1007/s00415-019-09683-5 31897600

[59] Valeriani D Simonyan K. A microstructural neural network biomarker for dystonia diagnosis identified by a DystoniaNet deep learning platform. Proc Natl Acad Sci USA. (2020) 117:26398–405. 10.1073/pnas.2009165117 33004625 PMC7586425

[60] Reich MM Horn A Lange F Roothans J Paschen S Runge J. Probabilistic mapping of the antidystonic effect of pallidal neurostimulation: a multicentre imaging study. Brain. (2019) 142:1386–98. 10.1093/brain/awz046 30851091

[61] Okromelidze L Tsuboi T Eisinger RS Burns MR Charbel M Rana M. Functional and structural connectivity patterns associated with clinical outcomes in deep brain stimulation of the globus pallidus internus for generalized dystonia. AJNR Am J Neuroradiol. (2020) 41:508–14. 10.3174/ajnr.A6429 32054614 PMC7077906

[62] Battistella G Fuertinger S Fleysher L Ozelius LJ Simonyan K. Cortical sensorimotor alterations classify clinical phenotype and putative genotype of spasmodic dysphonia. Eur J Neurol. (2016) 23:1517–27. 10.1111/ene.13067 27346568 PMC5308055

[63] Li Z Prudente CN Stilla R Sathian K Jinnah HA Hu X. Alterations of resting-state fMRI measurements in individuals with cervical dystonia. Hum Brain Mapp. (2017) 38:4098–108. 10.1002/hbm.23651 28504361 PMC5553075

[64] Albrecht F Mueller K Ballarini T Lampe L Diehl-Schmid J Fassbender K. Unraveling corticobasal syndrome and alien limb syndrome with structural brain imaging. Cortex. (2019) 117:33–40. 10.1016/j.cortex.2019.02.015 30927559

[65] Gonzalez-Escamilla G Muthuraman M Reich MM Koirala N Riedel C Glaser M. Cortical network fingerprints predict deep brain stimulation outcome in dystonia. Mov Disord. (2019) 34:1537–46. 10.1002/mds.27808 31433874

[66] Shah SA Brown P Gimeno H Lin J-P McClelland VM. Application of machine learning using decision trees for prognosis of deep brain stimulation of globus pallidus internus for children with dystonia. Front Neurol. (2020) 11:825. 10.3389/fneur.2020.00825 32849251 PMC7115974

[67] Fiorio M Gambarin M Valente EM Liberini P Loi M Cossu G. Defective temporal processing of sensory stimuli in DYT1 mutation carriers: a new endophenotype of dystonia? Brain. (2007) 130:134–42. 10.1093/brain/awl283 17105745

[68] Sadnicka A Daum C Meppelink A-M Manohar S Edwards M. Reduced drift rate: a biomarker of impaired information processing in functional movement disorders. Brain. (2020) 143:674–83. 10.1093/brain/awz387 31865371

[69] Hutchinson M McGovern EM Narasimham S Beck R Reilly RB Walsh CD. The premotor syndrome of cervical dystonia: disordered processing of salient environmental stimuli. Mov Disord. (2018) 33:232–7. 10.1002/mds.27229 29205495

[70] Kendler KS Neale MC. Endophenotype: a conceptual analysis. Mol Psychiatry. (2010) 15:789–97. 10.1038/mp.2010.8 20142819 PMC2909487

[71] Bradley D Whelan R Walsh R Reilly RB Hutchinson S Molloy F. Temporal discrimination threshold: VBM evidence for an endophenotype in adult onset primary torsion dystonia. Brain. (2009) 132:2327–35. 10.1093/brain/awp156 19525326

[72] Kimmich O Molloy A Whelan R Williams L Bradley D Balsters J. Temporal discrimination, a cervical dystonia endophenotype: penetrance and functional correlates. Mov Disord. (2014) 29:804–11. 10.1002/mds.25822 24482092

[73] Mc Govern EM Killian O Narasimham S Quinlivan B Butler JB Beck R. Disrupted superior collicular activity may reveal cervical dystonia disease pathomechanisms. Sci Rep. (2017) 7:16753. 10.1038/s41598-017-17074-x 29196716 PMC5711841

[74] Adolphs R. The social brain: neural basis of social knowledge. Annu Rev Psychol. (2009) 60:693–716. 10.1146/annurev.psych.60.110707.163514 18771388 PMC2588649

[75] Rinnerthaler M Benecke C Bartha L Entner T Poewe W Mueller J. Facial recognition in primary focal dystonia. Mov Disord. (2006) 21:78–82. 10.1002/mds.20659 16114021

[76] Czekóová K Zemánková P Shaw DJ Bareš M. Social cognition and idiopathic isolated cervical dystonia. J Neural Transm. (2017) 124:1097–104. 10.1007/s00702-017-1725-8 28444457

[77] Burke T Monaghan R McCormack D Al E. Cognition & social cognition in cervical dystonia. Clin Park ARD. (2020) 3:100072. 10.1016/j.prdoa.2020.100072 PMC8298799 34316651

[78] Ellement B Jasaui Y Kathol K Nosratmirshekarlou E Pringsheim T Sarna J. Social cognition in cervical dystonia: phenotype and relationship to anxiety and depression. Eur J Neurol. (2021) 28:98–107. 10.1111/ene.14508 32896024

[79] Foley JA Vinke RS Limousin P Cipolotti L. Relationship of cognitive function to motor symptoms and mood disorders in patients with isolated dystonia. Cogn Behav Neurol Off J Soc Behav Cogn Neurol. (2017) 30:16–22. 10.1097/WNN.0000000000000117 28323682

[80] Bleton J-P. Physiotherapy of focal dystonia: a physiotherapist's personal experience. Eur J Neurol. (2010) 17:107–12. 10.1111/j.1468-1331.2010.03061.x 20590817

[81] van den Dool J Visser B Koelman JH Engelbert RH Tijssen MA. Long-term specialized physical therapy in cervical dystonia: outcomes of a randomized controlled trial. Arch Phys Med Rehabil. (2019) 100:1417–25. 10.1016/j.apmr.2019.01.013 30796919

[82] Gimeno H Brown RG Lin J-P Cornelius V Polatajko HJ. Cognitive approach to rehabilitation in children with hyperkinetic movement disorders post-DBS. Neurology. (2019) 92:e1212–24. 10.1212/WNL.0000000000007092 30796136 PMC6511104

[83] Contarino MF Smit M van den Dool J Volkmann J Tijssen MAJ. Unmet needs in the management of cervical dystonia. Front Neurol. (2016) 7:165. 10.3389/fneur.2016.00165 27733842 PMC5039169

[84] Hefter H Rosenthal D Moll M. High botulinum toxin-neutralizing antibody prevalence under long-term cervical dystonia treatment. Mov Disord Clin Pract. (2016) 3:500–6. 10.1002/mdc3.12322 30363520 PMC6178717

[85] Albrecht P Jansen A Lee J-I Moll M Ringelstein M Rosenthal D. High prevalence of neutralizing antibodies after long-term botulinum neurotoxin therapy. Neurology. (2019) 92:e48–54. 10.1212/WNL.0000000000006688 30464031

[86] Hong JS Sathe GG Niyonkuru C Munin MC. Elimination of dysphagia using ultrasound guidance for botulinum toxin injections in cervical dystonia. Muscle Nerve. (2012) 46:535–9. 10.1002/mus.23409 22987694

[87] Koy A Lin J-P Sanger TD Marks WA Mink JW Timmermann L. Advances in management of movement disorders in children. Lancet Neurol. (2016) 15:719–35. 10.1016/S1474-4422(16)00132-0 27302239

[88] Krauss JK Pohle T Weber S Ozdoba C Burgunder JM. Bilateral stimulation of globus pallidus internus for treatment of cervical dystonia. Lancet. (1999) 354:837–8. 10.1016/S0140-6736(99)80022-1 10485734

[89] Kumar R Dagher A Hutchison WD Lang AE Lozano AM. Globus pallidus deep brain stimulation for generalized dystonia: clinical and PET investigation. Neurology. (1999) 53:871–4. 10.1212/WNL.53.4.871 10489059

[90] Coubes P Roubertie A Vayssiere N Hemm S Echenne B. Treatment of DYT1-generalised dystonia by stimulation of the internal globus pallidus. Lancet. (2000) 355:2220–1. 10.1016/S0140-6736(00)02410-7 10881900

[91] Kaminska M Perides S Lumsden DE Nakou V Selway R Ashkan K. Complications of deep brain stimulation (DBS) for dystonia in children - the challenges and 10 year experience in a large paediatric cohort. Eur J Paediatr Neurol EJPN Off J Eur Paediatr Neurol Soc. (2017) 21:168–75. 10.1016/j.ejpn.2016.07.024 27567277

[92] Moro E LeReun C Krauss JK Albanese A Lin J-P Walleser Autiero S. Efficacy of pallidal stimulation in isolated dystonia: a systematic review and meta-analysis. Eur J Neurol. (2017) 24:552–60. 10.1111/ene.13255 28186378 PMC5763380

[93] Volkmann J Mueller J Deuschl G Kühn AA Krauss JK Poewe W. Pallidal neurostimulation in patients with medication-refractory cervical dystonia: a randomised, sham-controlled trial. Lancet Neurol. (2014) 13:875–84. 10.1016/S1474-4422(14)70143-7 25127231

[94] Contarino MF Krauss JK. Dystonia. In: Temel Y Leentjens A de Bie R Chabardes S Fasano A. Fundamentals and Clinics of Deep Brain Stimulation - An Interdisciplinary Approach. Cham: Springer (2020). p. 217–234. 10.1007/978-3-030-36346-8

[95] Krauss J Lipsman N Aziz T Boutet A Brown P Chang J. Technology of deep brain stimulation: current state and future directions. Nat Rev Neurol. (2020) 17:75–87. 10.1038/s41582-020-00426-z 33244188 PMC7116699

[96] Capelle H-H Blahak C Schrader C Baezner H Hariz MI Bergenheim T. Bilateral deep brain stimulation for cervical dystonia in patients with previous peripheral surgery. Mov Disord. (2012) 27:301–4. 10.1002/mds.24022 22173964

[97] Meng Y Suppiah S Scantlebury N Lipsman N Schwartz ML. Treatment of a patient with task-specific writing tremor using magnetic resonance-guided focused ultrasound. Can J Neurol Sci Le J Can des Sci Neurol. (2018) 45:474–7. 10.1017/cjn.2018.19 29734963

[98] Saryyeva A Capelle H Kinfe H Schrader C Krauss J. Pallidal deep brain stimulation in patients with prior bilateral pallidotomy and selective denervation for treatment of dystonia. Stereotact Funct Neurosurg. (2020) 99:1–5. 10.1159/000509822 33080617

[99] Contarino MF Van Den Munckhof P Tijssen MAJ de Bie RMA Bosch DA Schuurman PR. Selective peripheral denervation: comparison with pallidal stimulation and literature review. J Neurol. (2014) 261:300–8. 10.1007/s00415-013-7188-4 24257834

[100] Tisch S. Recent advances in understanding and managing dystonia. F1000Research. (2018) 7:13823. 10.12688/f1000research.13823.1 30079230 PMC6058462

[101] Oterdoom DLM van Egmond ME Ascencao LC van Dijk JMC Saryyeva A Beudel M. Reversal of status dystonicus after relocation of pallidal electrodes in dyt6 generalized dystonia. Tremor Other Hyperkinet Mov (N Y). (2018) 8:530. 10.5334/tohm.409 29520331 PMC5840317

[102] Gimeno H Lin J-P. The International Classification of Functioning (ICF) to evaluate deep brain stimulation neuromodulation in childhood dystonia-hyperkinesia informs future clinical & research priorities in a multidisciplinary model of care. Eur J Paediatr Neurol EJPN Off J Eur Paediatr Neurol Soc. (2017) 21:147–67. 10.1016/j.ejpn.2016.08.016 27707656

[103] Valadas A Contarino M-F Albanese A Bhatia KP Falup-Pecurariu C Forsgren L. Management of dystonia in Europe: a survey of the European network for the study of the dystonia syndromes. Eur J Neurol. (2016) 23:772–9. 10.1111/ene.12940 26826067

[104] Koy A Weinsheimer M Pauls KAM Kühn AA Krause P Huebl J. German registry of paediatric deep brain stimulation in patients with childhood-onset dystonia (GEPESTIM). Eur J Paediatr Neurol EJPN Off J Eur Paediatr Neurol Soc. (2017) 21:136–46. 10.1016/j.ejpn.2016.05.023 27424797

[105] Artusi CA Dwivedi A Romagnolo A Bortolani S Marsili L Imbalzano G. Differential response to pallidal deep brain stimulation among monogenic dystonias: systematic review and meta-analysis. J Neurol Neurosurg Psychiatry. (2020) 91:426–33. 10.1136/jnnp-2019-322169 32079672

[106] Kroneberg D Plettig P Schneider G-H Kühn AA. Motor cortical plasticity relates to symptom severity and clinical benefit from deep brain stimulation in cervical dystonia. Neuromodulation. (2018) 21:735–40. 10.1111/ner.12690 28961350

[107] Lumsden DE Ashmore J Ball G Charles-Edwards G Selway R Ashkan K. Fractional anisotropy in children with dystonia or spasticity correlates with the selection for DBS or ITB movement disorder surgery. Neuroradiology. (2016) 58:401–8. 10.1007/s00234-015-1639-9 26759316 PMC4819774

[108] McClelland VM Fialho D Flexney-Briscoe D Holder GE Elze MC Gimeno H. Somatosensory Evoked Potentials and Central Motor Conduction Times in children with dystonia and their correlation with outcomes from Deep Brain Stimulation of the Globus pallidus internus. Clin Neurophysiol Off J Int Fed Clin Neurophysiol. (2018) 129:473–86. 10.1016/j.clinph.2017.11.017 29254860 PMC5786451

[109] Gundel H Wolf A Xidara V Busch R Ceballos-Baumann AO. Social phobia in spasmodic torticollis. J Neurol Neurosurg Psychiatry. (2001) 71:499–504. 10.1136/jnnp.71.4.499 11561034 PMC1763525

[110] Klingelhoefer L Chaudhuri KR Kamm C Martinez-Martin P Bhatia K Sauerbier A. Validation of a self-completed dystonia non-motor symptoms questionnaire. Ann Clin Transl Neurol. (2019) 6:2054–65. 10.1002/acn3.50900 31560179 PMC6801169

[111] Ribot B Aupy J Vidailhet M Mazere J Pisani A Bezard E. Dystonia and dopamine: from phenomenology to pathophysiology. Prog Neurobiol. (2019) 182:101678. 10.1016/j.pneurobio.2019.101678 31404592

[112] Smit M Bartels AL van Faassen M Kuiper A Niezen-Koning KE Kema IP. Serotonergic perturbations in dystonia disorders-a systematic review. Neurosci Biobehav Rev. (2016) 65:264–75. 10.1016/j.neubiorev.2016.03.015 27073048

[113] Zoons E Booij J Delnooz CCS Dijk JM Dreissen YEM Koelman JHTM. Randomised controlled trial of escitalopram for cervical dystonia with dystonic jerks/tremor. J Neurol Neurosurg Psychiatry. (2018) 89:579–85. 10.1136/jnnp-2017-317352 29326295

[114] Gimeno H Gordon A Tustin K Lin J-P. Functional priorities in daily life for children and young people with dystonic movement disorders and their families. Eur J Paediatr Neurol EJPN Off J Eur Paediatr Neurol Soc. (2013) 17:161–8. 10.1016/j.ejpn.2012.07.007 22889754

[115] Lumsden DE Gimeno H Tustin K Kaminska M Lin J-P. Interventional studies in childhood dystonia do not address the concerns of children and their carers. Eur J Paediatr Neurol EJPN Off J Eur Paediatr Neurol Soc. (2015) 19:327–36. 10.1016/j.ejpn.2015.01.003 25661063

[116] Eggink H Toonen RF van Zijl JC van Egmond ME Bartels AL Contarino MF. The effectiveness of deep brain stimulation in dystonia: a patient-centered approach. Tremor Other Hyperkinet Mov (N Y). (2020) 10:2. 10.5334/tohm.69 32775016 PMC7394190

[117] Gimeno H Tustin K Lumsden D Ashkan K Selway R Lin J-P. Evaluation of functional goal outcomes using the Canadian Occupational Performance Measure (COPM) following Deep Brain Stimulation (DBS) in childhood dystonia. Eur J Paediatr Neurol EJPN Off J Eur Paediatr Neurol Soc. (2014) 18:308–16. 10.1016/j.ejpn.2013.12.010 24461258

[118] Hariz G-M Limousin P Tisch S Jahanshahi M Fjellman-Wiklund A. Patients' perceptions of life shift after deep brain stimulation for primary dystonia–a qualitative study. Mov Disord. (2011) 26:2101–6. 10.1002/mds.23796 21626564

[119] Scaratti C Zorzi G Guastafierro E Leonardi M Covelli V Toppo C. Long term perceptions of illness and self after Deep Brain Stimulation in pediatric dystonia: a narrative research. Eur J Paediatr Neurol EJPN Off J Eur Paediatr Neurol Soc. (2020) 26:61–7. 10.1016/j.ejpn.2020.02.010 32147411

[120] van Egmond ME Eggink H Kuiper A Sival DA Verschuuren-Bemelmans CC Tijssen MAJ. Crossing barriers: a multidisciplinary approach to children and adults with young-onset movement disorders. J Clin Mov Disord. (2018) 5:3. 10.1186/s40734-018-0070-x 29636982 PMC5887190

[121] Scherbaum R Hartelt E Kinkel M Gold R Muhlack S Tonges L. Parkinson's Disease Multimodal Complex Treatment improves motor symptoms, depression and quality of life. J Neurol. (2020) 267:954–65. 10.1007/s00415-019-09657-7 31797086

